# Anwendungspraktische Limitationen bei der Aufklärung im Rahmen von Arzneimitteltherapien

**DOI:** 10.1007/s00063-021-00856-7

**Published:** 2021-09-01

**Authors:** Thomas Meyer, Melanie Steuer

**Affiliations:** 1 Klinik für Psychosomatische Medizin und Psychotherapie, Medizinische Fakultät, Georg-August-Universität Göttingen, Waldweg 33, 37073 Göttingen, Deutschland; 2 Deutsches Zentrum für Herz-Kreislauf-Forschung (DZHK), Standort Göttingen, Göttingen, Deutschland; 3 Institut für Kriminalwissenschaften, Abteilung für strafrechtliches Medizin- und Biorecht, Juristische Fakultät, Georg-August-Universität Göttingen, Göttingen, Deutschland

**Keywords:** Pharmakotherapie, Aufklärungspflicht, Unerwünschte Arzneimittelwirkungen, Recht auf Nichtwissen, Arzthaftung, Drug therapy, Information, Adverse drug effects, Right to non-knowledge, Medical liability

## Abstract

Der Erfolg einer Pharmakotherapie wird durch das Auftreten unerwünschter Arzneimittelwirkungen und die nur schwer vorhersehbaren Interaktionen zwischen mehreren Arzneimitteln bei Polypharmazie begrenzt. Bei Berücksichtigung der komplexen Wirkungsweise von Arzneimitteln einschließlich ihrer jeweils spezifischen Nebenwirkungsprofile werden an das ärztliche Aufklärungsgespräch mit Blick auf das Gebot einer patientengerechten Verständlichkeit hohe Anforderungen gestellt, insbesondere in Bezug auf eine ordnungsgemäße und vollständige Durchführung der Risiko- und Alternativaufklärung, aber auch hinsichtlich der Sicherungsaufklärung. Doch diese Anforderungen lassen sich praktisch wohl kaum jemals im medizinischen Alltag wirklich umsetzen. In diesem Artikel sollen deshalb anhand ausgewählter, aktueller Rechtsprechung die anwendungspraktischen Limitationen bei der Aufklärung vor der Einleitung und Überwachung einer Arzneimitteltherapie diskutiert werden. Im Besonderen wird auf bestehende Konfliktpotenziale zwischen dem Patientenrechtegesetz und dem sogenannten „Recht auf Nichtwissen“ hingewiesen.

## Abstract (en)

The efficacy of pharmacotherapy is limited by the occurrence of adverse drug reactions and the interactions between several drugs in polypharmacy, which are difficult to predict. Taking into account the complex mode of action of drugs, including their specific profiles of side effects, high demands are placed on the medical information with respect to a proper and complete clarification of the risks and alternative information under the precept of patient-oriented comprehensibility; however, these requirements can hardly ever be fulfilled in everyday medical practice. This article discusses the practical limitations of the information provided prior to the initiation and monitoring of drug therapy on the basis of selected current case law. In particular, the potential for conflict between the Patients’ Rights Act and the so-called right to non-knowledge is highlighted.

## Häufigkeit von tödlichen und nichttödlichen unerwünschten Arzneimittelwirkungen

Mit den Erfolgen der pharmakologischen Grundlagenforschung und ihrer Verankerung in der klinischen Pharmakologie haben sich die therapeutischen Möglichkeiten einer Arzneimittelbehandlung zweifellos verbessert. Viele Bereiche der Medizin, insbesondere der Transplantationsmedizin und Onkologie, aber auch der modernen kardiovaskulären Medizin, haben von den Fortschritten der Pharmakologie erheblich profitiert und sind ohne die Errungenschaften der modernen Arzneimitteltherapie heutzutage nicht mehr vorstellbar. Bei gezielter Indikationsstellung stehen für viele Erkrankungen hochwirksame Medikamente in einem Umfang zur Verfügung, der bei sorgfältiger und bestimmungsgemäßer Anwendung verbesserte Behandlungsstrategien verspricht, die noch vor wenigen Dekaden in dieser Form so nicht absehbar waren. Mit den Fortschritten der Arzneimitteltherapie ergeben sich allerdings naturgemäß auch nicht zu vernachlässigende, inhärente Risiken, die insbesondere bei bestimmungsgemäßem Gebrauch, und nicht bloß bei offensichtlichen Dosierungsfehlern oder Nichtbeachten von Kontraindikationen, den Behandlungserfolg gefährden können. Deshalb bedarf jede Entscheidung für eine Pharmakotherapie einer gründlichen Indikationsstellung unter Beachtung einer individualisierten Nutzenabwägung, bei der der vermutete therapeutische Gewinn für den Erkrankten gegenüber der Wahrscheinlichkeit von unter Umständen sogar tödlichen unerwünschten Arzneimittelwirkungen abgewogen werden muss.

Dabei ist es grundsätzlich angebracht, zwischen unerwünschten Arzneimittelwirkungen (UAW), die bei vorhandener Indikation und richtig gewählter Dosierung als unbeabsichtigte, schädliche Nebenwirkungen auftreten können, und unerwünschten Arzneimittelereignissen (UAE) als schädlichen, unvorhersehbaren Reaktionen bei Anwendung eines Arzneimittels zu unterscheiden [1]. Die Häufigkeit des Auftretens unerwünschter Arzneimittelreaktionen steigt mit der Anzahl der verabreichten Medikamente und bei einer Verordnung außerhalb des von den Arzneimittelbehörden zugelassenen Gebrauchs; namentlich pädiatrische und geriatrische Patienten sind bei zulassungsüberschreitender Anwendung einem hohen Risiko für unerwünschte Arzneimittelwirkungen ausgesetzt [2–6]. Auch wenn die Off-Label-Anwendung prinzipiell durch die ärztliche Therapiefreiheit abgesichert ist, werden an die sachgemäße Durchführung der Aufklärung erhöhte Anforderungen gestellt, wobei der Patient stets über den Versuchscharakter des Therapieversuchs unterrichtet werden muss [7].

Aufgrund einer unzureichenden Datenlage existieren keine verlässlichen Angaben zu den Häufigkeiten des Auftretens von schwerwiegenden Arzneimittelereignissen; dies gilt sowohl für die bestimmungsgemäße Durchführung einer Arzneimitteltherapie als auch für prinzipiell vermeidbare, iatrogene Medikationsfehler etwa im Zusammenhang mit unüblichen Dosierungen außerhalb des empfohlenen therapeutischen Bereichs [8]. Dennoch wird davon ausgegangen, dass das Auftreten von unerwünschten Arzneimittelwirkungen sowie von vermeidbaren ärztlichen Fehlern bei der Indikationsstellung und der Durchführung einer Pharmakotherapie häufige Ursachen von Krankenhauseinweisungen sind, die maßgeblich zur Krankenhausmortalität beitragen [1, 9].

Eine norwegische Studie führte 18,2 % (133 von insgesamt 732) aller Todesfälle in einer internistischen Abteilung eines überregionalen Krankenhauses ursächlich oder mitbedingt auf tödliche unerwünschten Arzneimittelereignisse zurück, wobei mit höherem Lebensalter und dem Vorliegen von Komorbiditäten und Polypharmazie die Häufigkeit fataler Arzneimittelreaktionen generell zunahm [10, 11]. Eine retrospektive Analyse von 1708 stationären Todesfällen im Universitätsklinikum Helsinki während des Jahres 2012 ergab bei 52 der Verstorbenen (3,0 %) einen sicheren oder wahrscheinlichen Hinweis auf eine tödliche arzneimittelinduzierte Reaktion und bei weiteren 24 Patienten (1,4 %) konnte eine mögliche Mitbedingtheit durch ein fatales Arzneimittelereignis vorgelegen haben [12]. Eine Studie aus dem Universitätsklinikum Erlangen ergab, dass in 6,2 % der Fälle von Ersteinweisungen und in 4,2 % der Wiedereinweisungen unerwünschte Arzneimittelreaktionen ursächlich verantwortlich waren [13]. Bei der Auswertung von Querschnittsdaten aus dänischen Krankenhäusern kommen Tchijevitch und Kollegen zu einer Zahl von 7,6 % der unerwartet auf Intensivstationen verlegten Patienten (8 von 105 Patienten), bei denen als Ursache ein potenziell lebensbedrohlicher Zustand durch ein unerwartetes Arzneimittelereignis vorlag. Bei insgesamt 5,5 % der Todesfälle (2 von 36 Patienten) wurde ein tödliches Arzneimittelereignis vermutet [14]. In einer Auswertung von Daten eines regionalen Pharmakovigilanznetzwerkes gehen Rottenkolber und Koautoren von einer geschätzten Inzidenz von 3,25 % vermuteter oder gesicherter schwerwiegender Arzneimittelreaktionen aus und beziffern die jährlichen volkswirtschaftlichen Kosten, die dadurch für Deutschland anfallen, auf eine Summe von insgesamt 434 Mio. € [15]. In der zwischen 2012 und 2018 durchgeführten, italienischen MEREAFaPS-Studie wurden bei 337 arzneimittelinduzierten Todesfällen zu 40 % Antikoagulanzien oder Thrombozytenfunktionshemmer als ursächlich angesehen [16]. Eine gleichfalls hohe Inzidenz von unerwünschten Arzneimittelreaktionen aus der ATC-Klasse (anatomisch-therapeutisch-chemische Einordnung) der Antithrombotika fand sich neben der von Zytostatika auch in einer retrospektiven, monozentrischen Studie, die im Jahr 2018 in der Notaufnahme des Universitätsklinikums Helsinki durchgeführt wurde [17]. Zu ähnlichen Ergebnissen kommen auch Daten aus den portugiesischen und deutschen Pharmakovigilanzsystemen bei älteren Patienten sowie Studienergebnisse aus Metaanalysen [18–21].

## Schwierigkeiten der Sicherungs- und Selbstbestimmungsaufklärung bei der Pharmakotherapie

Angesichts der besorgniserregenden Inzidenz von unerwünschten Arzneimittelreaktionen und deren Bedeutung für den klinischen Alltag ist es ein wenig überraschend, dass Arzthaftungsprozesse überwiegend aus anderen Gründen denn wegen unzureichender oder fehlerhafter Aufklärung über mögliche Arzneimittelwirkungen geführt werden. Ob sich diese Diskrepanz mit der tatsächlichen ärztlichen Fehlerrate im klinischen Alltag deckt oder inwieweit hierbei Verzerrungen bei der patientenseitigen Wahrnehmung von Aufklärungsfehlern zum Tragen kommen, lässt sich aufgrund fehlender Datenlage natürlich nicht beantworten. Dennoch ist sicherlich der Einschätzung zuzustimmen, dass mehr Erkrankte durch die Nichtverordnung einer an sich medizinisch notwendigen Medikation zu Schaden gelangen als im umgekehrten Fall durch die Verschreibung eines indizierten Arzneimittels, bei dem es im Verlauf der Behandlung zu nicht absehbaren Komplikationen kommt [22].

Eine Rolle mag in diesem Zusammenhang spielen, dass pharmakologische Behandlungsstrategien oftmals nicht unmittelbar nach Beginn der Medikation, sondern erst schleichend und zeitlich versetzt die gewünschten Effekte, aber eben auch unerwünschte Reaktionen hervorbringen können, sodass für den Einzelfall ein kausaler Zusammenhang aus der Perspektive des Betroffenen oder seiner Angehörigen oftmals nicht ersichtlich wird. Die Verschlechterung des klinischen Zustands wird dabei vom Patienten nicht als eine unbeabsichtigte Reaktion auf ein zuvor verordnetes Medikament in Verbindung gebracht, sondern häufig als eine unbeeinflussbare Progression der Grunderkrankung fehlgedeutet, bei der die Medikation eben nicht angeschlagen habe. Zudem ist die Wirkung einer pharmakologischen Intervention weniger anschaulich vermittelbar als die sich besser vorstellbaren Effekte eines kurativen, operativen Eingriffs, der etwa auf die Exstirpation eines erkrankten Organs oder die Rekonstruktion einer verletzten anatomischen Struktur abzielt.

Aufgrund fehlender Vorkenntnisse müssen dem medizinischen Laien die molekularen Angriffspunkte einer Pharmakotherapie oftmals unverständlich bleiben und auch in der Arzneimittelinformation in der Packungsbeilage werden die Wirkungen nicht mit der gebührlichen Ausführlichkeit und Laienverständlichkeit thematisiert, sofern der Beipackzettel unter stationären Bedingungen überhaupt ausgehändigt wird, was wohl selten der Fall sein dürfte. Die aufgezeigte Problematik der sog. Sicherungsaufklärung i. S. d. § 630c Abs. 1 S. 1 BGB hat darüber hinaus aber auch Auswirkungen auf die Selbstbestimmungsaufklärung, denn bedingt durch die Komplexität der Therapie werden an die eigenverantwortliche und informierte Entscheidungsfindung des Patienten über den erhofften Nutzen einer Arzneimitteltherapie hohe Anforderungen gestellt. Zwischen immer umfangreicheren Aufklärungsverpflichtungen und der Vermittlung medizinischer Komplexität droht der Anspruch einer normativ eingeforderten, gemeinsamen Entscheidungsfindung von Arzt und Patienten zu zerbrechen [23].

Nachfolgend sollen daher anhand der aktuellen Gesetzeslage die Grenzen der ärztlichen Aufklärungspflicht bei der Einleitung und Überwachung einer medikamentösen Therapie skizziert und der Frage nachgegangen werden, wie die dahingehende Aufklärungsverpflichtung durch die aktuelle Rechtsprechung in Anbetracht seltener, aber schwerwiegender Komplikationen einer außerhalb von klinischen Studien stattfindenden Pharmakotherapie praktisch ausgelegt wird.

## Reichweite und Grenzen der Aufklärungspflichten im Rahmen der Pharmakotherapie

Der Verordnung und Anwendung eines Arzneimittels muss zwingend eine adäquate, sachgerechte Aufklärung über die Arzneimitteltherapie vorausgehen, wobei diese generell patienten- und situationsbezogen erfolgen muss [24]. Nach Maßgabe des § 630e Abs. 1 BGB muss das Aufklärungsgespräch dem Patienten alle entscheidenden Informationen und Grundlagen liefern, damit dieser eine rationale Entscheidung über die einzuschlagenden Therapieoptionen selbstbestimmt treffen kann. Die Aufklärungspflichten sind daher nach dem Wortsinn dieser Vorschrift sehr weit gefasst und schließen sämtliche behandlungsrelevanten Umstände wie Folgen und Risiken der konkreten Behandlung mit ein. Nach § 630e Abs. 1 S. 3 BGB ist der behandelnde Arzt neben der Risikoaufklärung bei der medikamentösen Therapie auch gehalten, den Patienten über Behandlungsalternativen mit wesentlich unterschiedlichen Risiken oder Erfolgsaussichten in Kenntnis zu setzen und ihm damit die Wahl, sofern dieser in der konkreten Situation eine echte Wahlmöglichkeit hat (BGH v. 22.09.1987 – VI ZR 238/86, NJW 1988, 763–764; OLG Naumburg v. 12.11.2009 – 1 U 59/09, NJW 2010, 1758–1759), zwischen den gleichermaßen medizinisch indizierten Behandlungsmethoden zu überlassen (BGH v. 22.02.2000 – VI ZR 100/99, NJW 2000, 1788–1789; BGH v. 15.03.2005 – VI ZR 313/03, NJW 2005, 1718; BGH v. 28.10.2014 – VI ZR 125/13, NJW-RR 2015, 591–592; BGH v. 03.09.2020 – 4 U 905/20, NJW-RR 2021, 25–26; zum Umfang der Aufklärung über nicht allgemein anerkannte Behandlungsmethoden vgl. die jüngste Entscheidung des BGH v. 15.10.2019 – VI ZR 105/18, NJW 2020, 1358; [25]). Fehlt es im konkreten Fall an einer an sich gegebenen Behandlungsalternative, so entfällt jedoch die Pflicht zur Aufklärung hierüber (OLG Oldenburg v. 19.12.2018 – 5 U 114/18, MDR 2019, 741).

Zur Vermeidung von Aufklärungsversäumnissen ist der Arzt daher getreu dem Ideal einer „vollständigen“ Informiertheit des Patienten grundsätzlich verpflichtet, den Patienten über sämtliche objektiv relevanten Fakten zwecks bestmöglicher Befähigung zur selbstbestimmten Entscheidung aufzuklären. Hierzu gehört es, den Patienten vor Beginn der Arzneimitteltherapie über die gewählte Dosis, die bekannten Unverträglichkeiten und die zu erwartenden Nebenwirkungen, die sich auch bei Anwendung der gebotenen Sorgfalt, also bei fehlerfreier Arzneimittelverordnung oder -anwendung, nicht mit Gewissheit ausschließen lassen, in Kenntnis zu setzen [26]. Dabei hat der aufklärende Arzt im Fall der medikamentösen Behandlung die Fachinformation nach § 11a AMG als eine wichtige Informationsquelle zu nutzen und darf davon ausgehen, dass diese nach fachlich-objektiven Maßstäben unter Einschluss aller veröffentlichten Ergebnisse und auch unveröffentlichten Meldungen über das Pharmakovigilanzsystem erstellt wurde (OLG Köln v. 21.03.2016 – 5 U 76/14, MedR 2017, 250–251).

Nach § 630h Abs. 2 S. 2 BGB kann der Arzt im Falle einer unzureichenden Aufklärung hinsichtlich des verwirklichten Risikos einer unerwünschten Arzneimittelreaktion geltend machen, dass der Patient auch bei ordnungsgemäßer Durchführung der Aufklärung in die Behandlung eingewilligt hätte. Damit das Selbstbestimmungsrecht des Patienten jedoch nicht untergraben wird, genügt insoweit der alleinige Verweis auf einen verständigen Patienten, der seine Einwilligung auch bei ausreichender Risikoaufklärung über die Gefährdungen durch die eingeleitete Medikation gegeben hätte, nicht für die Nachweiserbringung. Vielmehr bedarf es vonseiten des beklagten Arztes einer nachvollziehbaren Begründung, warum im konkreten Einzelfall der Geschädigte unter Beachtung seiner persönlichen Situation eine Zustimmung nicht verweigert hätte (vgl. bereits BGH v. 07.02.1984 – VI ZR 174/82, NJW 1984, 1397, 1399). Nach Auffassung des OLG Dresden trifft den Patienten auch bei nicht ordnungsgemäßer Aufklärung über den Off-Label-Gebrauch durch den beweisbelasteten Arzt die Darlegungs- und Beweislast hinsichtlich der Kausalität dieser aufklärungsbedingt rechtswidrigen Behandlung für den dauerhaften Gesundheitsschaden (OLG Dresden v. 15.05.2018 – 4 U 248/16, MedR 2018, 971–975; [27]).

Die Aufklärungsverpflichtung bei medikamentöser Therapie mit Benzodiazepinen schließt etwa die Erwähnung eines Suchtpotenzials bei chronischer Anwendung in Eigeneinnahme ein (OLG Dresden v. 07.06.2018 – 4 U 307/18, MedR 2019, 152–154). Regelmäßige Kontrolluntersuchungen als Verlaufsbeobachtung und zur Sicherung des therapeutischen Erfolgs sind nach Rechtsprechung des BGH zur Vermeidung von Behandlungsfehlern notwendig (BGH v. 27.03.2007 – VI ZR 55/05, MedR 2007, 653; [28]). Ein alleiniger Verweis auf den Beipackzettel des verordneten Medikaments ist aber nicht ausreichend, um den Vorwurf einer unterlassenen oder nicht ausreichenden Risiko- und Selbstbestimmungsaufklärung im Streitfall zu entkräften (BGH v. 15.03.2005 – VI ZR 289/03, NJW 2005, 1716–1718; [24]). Schließlich kann nur der Arzt selbst in Kenntnis der konkreten Situation des Patienten wissen, worauf es bei der Anwendung des von ihm verordneten Arzneimittels ankommt, während der Beipackzettel lediglich ein generelles Nutzen-Risiko-Profil darstellt.

## Häufigkeitsangaben zu Komplikationen im Aufklärungsgespräch

In weitgehender Übereinstimmung mit der medizinrechtlichen Literatur wurde in einem jüngeren Urteil des BGH (v. 29.01.2019 – VI ZR 117/18, NJW 2019, 1283–1284) bestätigt, dass sich die Wahrscheinlichkeitsangaben zu Behandlungskomplikationen in einer ordnungsgemäß durchgeführten ärztlichen Aufklärung nicht notwendig an den in der Gebrauchsinformation des Medikaments verwendeten Häufigkeitsdefinitionen des Medical Dictionary for Regulatory Activities zu orientieren haben, sondern davon abweichen können [29, 30]. Auf eine bestimmte Mindestwahrscheinlichkeit der Risikorealisierung soll es gerade nicht ankommen (so bereits BGH v. 23.10.1979 – VI ZR 197/78, NJW 1980, 633–634; OLG Koblenz v. 01.04.2004 – 5 U 844/03, MedR 2004, 501–502; OLG Köln v. 25.04.2007 – 5 U 180/05, VersR 2008, 1072; „selbst bei extrem seltenen Risiken“: BGHZ 126, 386–389: „Risikodichte, die sich nur im Promillebereich bewegt“). Der BGH bekräftigte 2009 die bisherige Rechtsprechung, dass über seltene oder äußerst seltene Komplikationen grundsätzlich dann aufgeklärt werden müsse, wenn sich diese zwar nur selten verwirklichen, aber hierdurch eine schwerwiegende Belastung für die zukünftige Lebensführung des Erkrankten resultiert (BGH v. 29.09.2009 – VI ZR 251/08, GesR 2010, 115).

Für die Risikogewichtung bei einer Pharmakotherapie liegt maßgeblich der konkrete Patientenbezug nach Einschätzung des Arztes zugrunde, nicht hingegen die Angaben aus einem statistischen Wahrscheinlichkeitsverständnis [31]. Eine einseitige Betonung der Häufigkeiten von Komplikationen und Risiken im Aufklärungsgespräch erscheint indes wenig zielführend, um den spezifischen Belangen einer individualisierten, am Einzelfall ausgerichteten Risikoaufklärung gerecht zu werden. Das Eingehen auf spezifische Risikowahrscheinlichkeiten, die sich aus den Umständen des Patienten und seinen Komorbiditäten herleiten lassen, bleibt entscheidend für die Umfänglichkeit einer Aufklärung über unerwünschte Arzneimittelreaktionen. Sofern ein gewisses medizinisches Vorwissen bei dem Patienten vorausgesetzt werden darf und unterstellt wird, dass er bei Verständigungsschwierigkeiten oder Unklarheiten im Aufklärungsgespräch den Mut zu weiteren Nachfragen aufbringen würde, kann sich der Arzt auf die pauschale Angabe von Komplikationen wie „Thrombose“ oder „Embolie“ beschränken, ohne diese dann im Detail erklären zu müssen [32].

## Aufklärung über Arzneimittelwirkungen begünstigt schädliche Noceboeffekte

Es wird in der ärztlichen Praxis wohl eher die seltene Ausnahme als die Regel sein, dass die Inhalte des vertrauensvollen Aufklärungsgesprächs zwischen behandelndem Arzt und dem Patienten in ihrer Umfänglichkeit über das hinausgehen, was die Fachinformation des pharmazeutischen Unternehmers an möglichen Nebenwirkungen aufführt. Im medizinischen Alltag berät der aufklärende Arzt über allgemein bekannte Arzneimittelwirkungen und gewichtet diese nach der Schwere der zu erwartenden Beeinträchtigung für seinen Patienten, wohlwissend dass sich Noceboeffekte bei umfänglicher Schilderung des Nebenwirkungsprofils negativ auf die Adhärenz zur Pharmakotherapie auswirken können. Die Fachinformation berücksichtigt demgegenüber auch solche Einzelfälle, bei denen nur von einer zeitlichen Koinzidenz von der Arzneimittelgabe und neu aufgetretenen klinischen Symptomen berichtet wurde, ein kausaler Zusammenhang aber nicht mit Sicherheit hergestellt werden konnte. In der medizinrechtlichen Literatur wurde mehrfach darauf hingewiesen, dass bei der Beurteilung von Aufklärungsfehlern durch Unterlassen zwar die entsprechende Fachinformation des pharmazeutischen Unternehmers als eine wichtige Informationsquelle durch den Patienten herangezogen werden sollte [24]. Im Streitfall ersetzt aber der Verweis auf die Fachinformation nicht die Hinzuziehung eines Sachverständigengutachtens über die konkrete patientenbezogene und haftungsbegründende Kausalität bei der unerwünschten Arzneimittelwirkung (OLG Köln v. 24.02.2016 – 5 U 77/15, MedR 2017, 148–150; OLG Köln v. 21.03.2016 – 5 U 76/14, MedR 2017, 250–251).

Eine Abweichung von der in Leitlinien üblicherweise verwendeten Dosierung sei dann nicht haftungsbegründend oder -ausfüllend, wenn der Evidenzgrad in der jeweils gültigen Leitlinie niedrig ist oder aber Wirkungshinweise auch bei der gewählten, abweichenden Dosierung in der medizinischen Literatur existieren (OLG Koblenz v. 04.11.2014 – 5 U 869/14, MedR 2015, 426–427; [33]). Für die juristische Einschätzung einer Haftungskonsequenz bei einer Abweichung von der gängigen Medikamentendosierung muss also in jedem Fall der medizinische Standard als maßgebliche Richtschnur zugrunde gelegt werden.

Wenn im laufenden Arzthaftungsprozess ein Behandlungsfehler nicht nachgewiesen werden kann, kommt es durchaus vor, dass von Klägerseite eine Aufklärungsfehlerrüge nachgeschoben wird. Dann dient die Geltendmachung eines Verstoßes gegen die ärztliche Aufklärungspflicht dazu, die Erfolgsaussichten einer Schadensersatzklage wegen Körperverletzung durch Verlagerung auf einen anderen Streitpunkt zu verbessern [34]. Das Einfordern von Schadensersatz durch eine unterstellte Verletzung der ärztlichen Sorgfaltspflicht bei der Umfänglichkeit der Aufklärungsinhalte stellt eine zivilrechtliche Prozessstrategie von Klägerseite dar, die durch die Reichweite des § 630e BGB erleichtert wird, wenngleich wohl eher in vielen Fällen ein schwerer beweisbarer Behandlungsfehler vermutet wird. Vom beklagten Arzt wird dann nicht selten die Rechtsfigur der hypothetischen Einwilligung bemüht, die besagt, dass der Patient auch dann der gewählten medizinischen Intervention zugestimmt hätte, wenn er zuvor hinreichend aufgeklärt worden wäre. Der Verweis auf die hypothetische Einwilligung führt aber unter forensischen Gesichtspunkten nur selten zu der gewünschten Entlastung, sondern verringert die Erfolgsaussichten einer Haftungsabwendung, da in diesem Fall der in die Beweislast gestellte Arzt darlegen muss, warum dem Patienten tatsächlich keine andere Wahl als die hypothetische Einwilligung übrigblieb. Der hypothetischen Einwilligung haftet deshalb schon von vornherein immer das Manko an, dass sie einer paternalistischen Vorstellung über die Arzt-Patienten-Beziehung folgt, die den Patienten in seiner freien Therapieentscheidung bevormundet und seine Autonomie zu selbstbestimmtem Handeln untergräbt [35].

## Auf der Suche nach anwendungsorientierten Lösungen für eine Reform des überzogenen Aufklärungsrechts

Das Prinzip der umfassenden (Selbstbestimmungs‑)Aufklärung, wie es im Patientenrechtegesetz in der im Jahre 2013 neu in das BGB eingefügten Vorschrift des § 630e hinterlegt ist, kollidiert im klinischen Alltag nicht selten mit dem gleichfalls unbestreitbaren „Recht auf Nichtwissen“. Die normative Geltungskraft eines „Rechts auf Nichtwissen“ – etwa die bewusste Entscheidung, über mögliche Komplikationen einer Medikamentengabe im Vorfeld einer Arzneimitteltherapie nicht vollumfänglich durch den Behandelnden informiert zu werden – beruht zweifelsohne auf einem berechtigten und schützenswürdigen Fundament der Patientenautonomie; doch scheint sich diese Erkenntnis erst allmählich gegen das Konstrukt des „informed consent“ durchzusetzen [36]. Unter zivilrechtlicher Perspektive stellt das sogenannte „Recht auf Nichtwissen“ – und eingeschlossen damit ein Recht auf Partialwissen – den aufklärenden Arzt vor die Herausforderung, sich auf die individuellen Aufklärungsbedürfnisse des Patienten vor Beginn einer Pharmakotherapie einstellen zu müssen und dabei zugleich mit den weitreichenden normativen Vorgaben des Patientenrechtegesetzes konform zu gehen. Eine probate Lösung zur Vermeidung von Haftungsansprüchen ist für die klinische Praxis hier vergleichsweise einfach und simpel: Der Arzt stellt seine Fürsorge in den Dienst einer Erfüllung der subjektiven Befindlichkeiten und Wünsche seines Patienten und pflegt einen authentischen und empathischen Kommunikationsstil, zugleich dokumentiert er die individuellen Aufklärungswünsche des Patienten. Wenn der Patient spürt, dass er im Mittelpunkt der ärztlichen Zuwendung steht, wird er es seiner Behandlerin oder seinem Behandler sicherlich positiv vergelten und keine Veranlassung für den Vorwurf einer ärztlichen Pflichtverletzung erkennen. Das zugewandte und offene Eingehen auf die individuellen Belange des Patienten ist damit die beste Rückversicherung gegenüber unberechtigten Regressforderungen und bewahrt den Arzt vor Haftungsklagen.

Die Einbeziehung des Patienten im Medikationsprozess ist von grundsätzlicher Bedeutung für die behandelnde Ärztin bzw. den behandelnden Arzt, um den Erkrankten über vermeidbare UAW zu sensibilisieren und um rechtzeitig entsprechende Gegenmaßnahmen ergreifen zu können. Mueller und Kirch sprechen in diesem Zusammenhang von „einer partnerschaftlichen Interaktion aller am Medikationsprozess Beteiligten“ [37], die für den längerfristigen Erfolg der Arzneimitteltherapie unabdingbar ist. Die situationsbezogene Aufklärung hat in einem vertrauensvollen, von Empathie getragenen Gespräch zu erfolgen; sie muss vor allem solche Risiken berücksichtigen, die beim Auftreten von UAW in die zukünftige Lebensgestaltung des Patienten eingreifen könnten. Dabei sollte der Arzt dem Wunsch des Patienten hinsichtlich des inhaltlichen Umfangs und der Tiefe der Aufklärung gebührend Rechnung tragen [38]. Eine Verpflichtung zur Verwendung bestimmter Dokumentationsformulare, wie sie etwa bei chirurgischen Operationen verwendet werden, gibt es bei der Pharmakotherapie nicht. Bezüglich der erforderlichen Dokumentationspflicht bestehen wenig konkrete Vorgaben, doch muss der Arzt im Streitfall glaubhaft darlegen können, über welche Risiken er den Patienten aufgeklärt hat.

Ungleich schwieriger aber als auf der medizinischen Ebene ist es, die strikten Vorgaben eines weitreichenden Patientenrechtegesetzes vor dem Hintergrund eines an Bedeutung zunehmenden „Rechts auf Nichtwissen“ auch auf einer juristischen Ebene unter anwendungspraktischen Gesichtspunkten verändern zu wollen. Denn die kritiklose Übernahme zivilrechtlicher Rechtsfiguren in das Strafrecht, wie es die einschlägige Rechtsprechung für die hypothetische Einwilligung gegenwärtig praktiziert, ist allein schon aus rechtsdogmatischer Sicht mehr als bedenklich. Dass also eine Notwendigkeit zur Anpassung der Gesetzeslage angesichts „der überzogenen Reichweite der ärztlichen Aufklärungspflicht“ [39] besteht, mag man kaum ernsthaft bezweifeln. Besonders offensichtlich wird dieses bei der Risikoaufklärung vor der Einleitung einer Pharmakotherapie, wo durch eine Vielzahl schwer abschätzbarer und unvorhersehbarer Komplikationen die negativen Folgen einer Defensivmedizin geradezu heraufbeschwört werden. Im Interesse eindeutiger Rechtssicherheit kommt der Wunsch nach einer Weiterentwicklung des Aufklärungsrechts im Sinne einer wahrhaften therapeutischen Partnerschaft damit beiden zugute: dem Patienten wie auch seinem ärztlichen Behandler.
